# Primary Neuron Culture for Nerve Growth and Axon Guidance Studies in Zebrafish (*Danio rerio*)

**DOI:** 10.1371/journal.pone.0057539

**Published:** 2013-03-04

**Authors:** Zheyan Chen, Han Lee, Steven J. Henle, Thomas R. Cheever, Stephen C. Ekker, John R. Henley

**Affiliations:** 1 Mayo Graduate School, College of Medicine, Mayo Clinic, Rochester, Minnesota, United States of America; 2 Department of Neurologic Surgery, Mayo Clinic, Rochester, Minnesota, United States of America; 3 Department of Biochemistry and Molecular Biology, Mayo Clinic, Rochester, Minnesota, United States of America; 4 Department of Physiology and Biomedical Engineering, Mayo Clinic, Rochester, Minnesota, United States of America; Harvard University, United States of America

## Abstract

Zebrafish (*Danio rerio*) is a widely used model organism in genetics and developmental biology research. Genetic screens have proven useful for studying embryonic development of the nervous system *in vivo*, but *in vitro* studies utilizing zebrafish have been limited. Here, we introduce a robust zebrafish primary neuron culture system for functional nerve growth and guidance assays. Distinct classes of central nervous system neurons from the spinal cord, hindbrain, forebrain, and retina from wild type zebrafish, and fluorescent motor neurons from transgenic reporter zebrafish lines, were dissociated and plated onto various biological and synthetic substrates to optimize conditions for axon outgrowth. Time-lapse microscopy revealed dynamically moving growth cones at the tips of extending axons. The mean rate of axon extension *in vitro* was 21.4±1.2 µm hr^−1^ s.e.m. for spinal cord neurons, which corresponds to the typical ∼0.5 mm day^−1^ growth rate of nerves *in vivo*. Fluorescence labeling and confocal microscopy demonstrated that bundled microtubules project along axons to the growth cone central domain, with filamentous actin enriched in the growth cone peripheral domain. Importantly, the growth cone surface membrane expresses receptors for chemotropic factors, as detected by immunofluorescence microscopy. Live-cell functional assays of axon extension and directional guidance demonstrated mammalian brain-derived neurotrophic factor (BDNF)-dependent stimulation of outgrowth and growth cone chemoattraction, whereas mammalian myelin-associated glycoprotein inhibited outgrowth. High-resolution live-cell Ca^2+^-imaging revealed local elevation of cytoplasmic Ca^2+^ concentration in the growth cone induced by BDNF application. Moreover, BDNF-induced axon outgrowth, but not basal outgrowth, was blocked by treatments to suppress cytoplasmic Ca^2+^ signals. Thus, this primary neuron culture model system may be useful for studies of neuronal development, chemotropic axon guidance, and mechanisms underlying inhibition of neural regeneration *in vitro,* and complement observations made *in vivo*.

## Introduction

The translucent nature of embryonic and larval zebrafish has enabled neurobiological research to be pursued *in vivo* with this model organism [1], [2], [3], [4], [5], [6], [7]. Moreover, forward genetic screens utilizing zebrafish have significantly increased our understanding of embryonic development of the nervous system [8], [9], [10]. However, unlike other widely used model organisms, *in vitro* studies utilizing zebrafish have been limited [11], [12], likely due to the suboptimal conditions and complexity of pioneering cell culture systems [13], [14].

### Strengths of the zebrafish model system

Zebrafish possess genetic tractability and physical advantages usually only found in invertebrate systems, yet have extensive conserved synteny to the human genome. They share most human genes, and gene functions are well conserved [15]. The strength of zebrafish genetics has been demonstrated by the high-resolution zebrafish genome comparable to that of human and mouse [10], the wide variety of zebrafish mutant phenotypes uncovered through large scale mutagenesis screens [16], and various genetic tools such as antisense morpholino oligonucleotides and transposons [17], [18], [19], [20]. Zebrafish have high fecundity, and an adult mating pair can produce hundreds of embryos per clutch per week. Embryonic development is complete within three to four days at room temperatures (22.5°–24°C) [21]. The average lifespan of zebrafish is 2–3 years, which is longer or equivalent to that of mice [22], and generation time is about 3 months [21].

*In vitro* studies using chick [23], frog [24], mouse [25], and rat [26] neurons have been essential in our understanding of neuronal cell biology and the molecular mechanisms underlying chemotropic guidance of growing axons [26], [27], [28], [29], [30]. Teleost retinal explants [31] have also been a useful tool to study axon guidance and regeneration in the eye [32], and zebrafish hindbrain explants allow dynamic analysis of neuronal migration *in vitro* [33]. Here we introduce a robust and easily adaptable zebrafish dissociated cell culture system for multiple classes of primary neurons. This culture system allowed us to perform functional assays of axon outgrowth and guidance in addition to high resolution live-cell Ca^2+^-imaging, and provide insight into Ca^2+^-dependent axon outgrowth. The approach is amendable to mechanistic studies of axon and dendrite development – including neuronal polarization, neurite initiation, outgrowth, axon guidance, synaptogenesis, and neural circuit formation – yet is also suitable for the classroom. Our method may complement not only the classic neuron culture systems utilizing chick, frog, mouse, and rat models, but also a recent method developed independently for the study of zebrafish motor neurons post-axogenesis [34].

## Results

### Axon guidance pathway homology

To assess the zebrafish genome for complements of human axon guidance genes, we carried out homology analyses of several important chemotropic factors (Table 1). We surveyed members of the Netrin [35], [36], Slit [36], Semaphorin [37], and Ephrin [38] classic axon guidance molecule families, and members of three prominent morphogen families that also act as axon guidance cues – Hedgehog [39], [40], Wnt [41], and TGFß/BMP [42]. We also investigated zebrafish homologs of the neurotrophic factors nerve growth factor and brain derived neurotrophic factor (BDNF), and the myelin component myelin-associated glycoprotein (MAG), which are involved in mammalian central nervous system injury and repair [43], [44]. Protein sequence comparisons revealed a 65.0% mean protein identity (range 28.3–86.7%), with a 97.1% mean protein coverage (range 90.5–100%) between human and zebrafish orthologs across regions of alignment. Thus, these pathways appear to be well conserved between humans and zebrafish.

**Table 1:** Human-zebrafish homology of axon guidance cues.

Human gene symbol	Human protein ID	ZF gene symbol	ZF protein ID	Protein Identity (%)	Protein Coverage (%)
NTN1	NP_004813.2	ntn1a	NP_571104.1	86.7	100.0
		ntn1b	NP_571073.1	82.1	99.7
SLIT2	NP_004778.1	slit2	NP_571810.1	79.9	99.2
SEMA3A	NP_006071.1	sema3aa	NP_571135.1	72.3	98.5
		sema3ab	NP_571136.1	75.3	96.6
EFNB3	NP_001397.1	efnb3a	NP_571172.1	28.3	97.8
		efnb3b	NP_571881.1	54.8	97.0
		LOC564234	XP_692670.2	67.8	99.1
SHH	NP_000184.1	shha	NP_571138.1	64.5	90.5
		shhb	NP_571274.2	68.2	94.7
WNT5B	NP_110402.2	wnt5b	NP_571012.1	80.5	97.5
BMP7	NP_001710.1	bmp7b	NP_001070614.1	79.0	96.0
BDNF	NP_733927.1	bdnf	NP_571670.2	69.6	91.5
NGF	NP_002497.2	ntf7	NP_571139.1	41.0	97.7
		ngf	NP_954680.1	49.0	99.7
MAG	NP_002352.1	mag	NP_001007063.1	41.2	98.1

Alignments were performed using NCBI BLAST (blastp). ZF: zebrafish. Protein identity: percentage of identical amino acids between the human and zebrafish orthologous proteins across the region of alignment. Protein coverage: percentage of human protein matched to orthologous zebrafish protein across the region of alignment.

### Characterization of primary spinal neuron cultures

**Figure 1:**
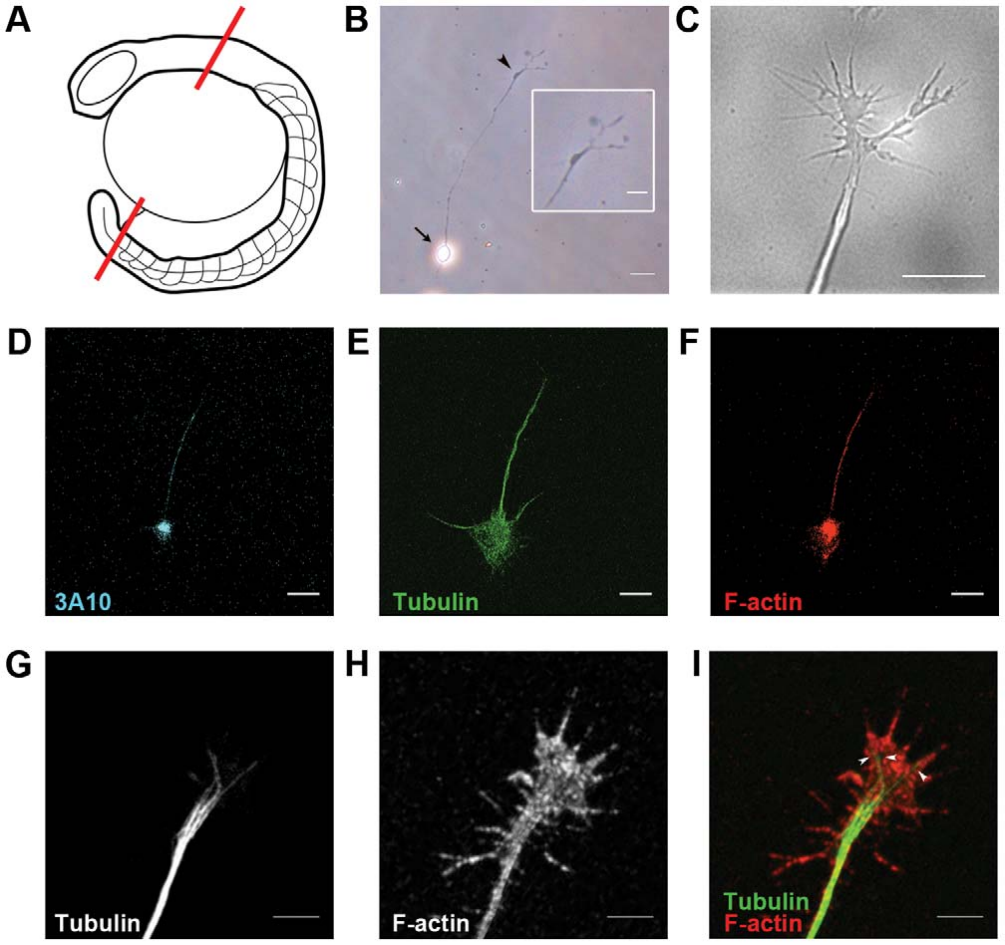
Characterization of zebrafish spinal neurons in culture. (A) Diagram of spinal cord dissection. Red lines indicate cut sites. (B) Dissociated spinal neuron in culture. Inset shows magnified view of growth cone with filopodia. Arrow: neuron cell body. Arrowhead: growth cone at axon tip. (C) Differential interference contrast live imaging shows filopodia and lamellipodia at the axon tip. Immunostaining for (D) neurofilament associated antigen, (E) tubulin, and (F) F-actin. Magnified views of growth cones reveal (G) bundled microtubules projecting along axons and terminating in the growth cone central domain, while (H) F-actin filaments are abundant in the peripheral filopodia and lamellipodia. (I) Merged image of growth cone cytoskeleton organization. Arrowheads: individual microtubules. Scale bars, 20 µm (B), 10 µm (B inset, D–F), 5 µm (C, G–I).

To study primary neurons from zebrafish *in vitro*, we devised a simple method to grow spinal neurons in culture. In this novel technique, we isolated developing spinal cords from zebrafish at the 20 somite stage (Fig. 1A), then enzymatically and mechanically dissociated the tissue into a uniform cell suspension. After plating the dissociated cells onto laminin-coated coverglass, cells started attaching to the substrate within 10 min and became fully adherent by 2 hr. By 3 hr after plating, neurons became polarized and started extending neurites, which by 6 hr developed into long axon extensions (Fig. 1B) tipped by growth cone expansions 1–5 µm in diameter (Fig. 1C). Growth cones in culture were highly dynamic, with constantly protruding and retracting filopodia and lamellipodia (Movie S1). The cultures also contained a mixed population of other cell types, morphologically resembling myoblasts and glial cells (Supporting Figure S1).

We investigated the cytoskeletal arrangements of zebrafish spinal neuron axons by immunocyotchemistry (Fig. 1D–I). Positive immunostaining for neurofilament associated antigen 3A10 was consistent with commissural interneuron staining in other species [30], [45] (Fig. 1D). Co-staining for tubulin and filamentous actin (F-actin) revealed that bundled microtubules projected along the entire length of the axon and largely terminated in the growth cone central domain (Fig. 1E, G, I), whereas F-actin extended along the axon and was relatively enriched in the growth cone peripheral filopodia and lamellipodia (Fig. 1F, H, I).

**Figure 2:**
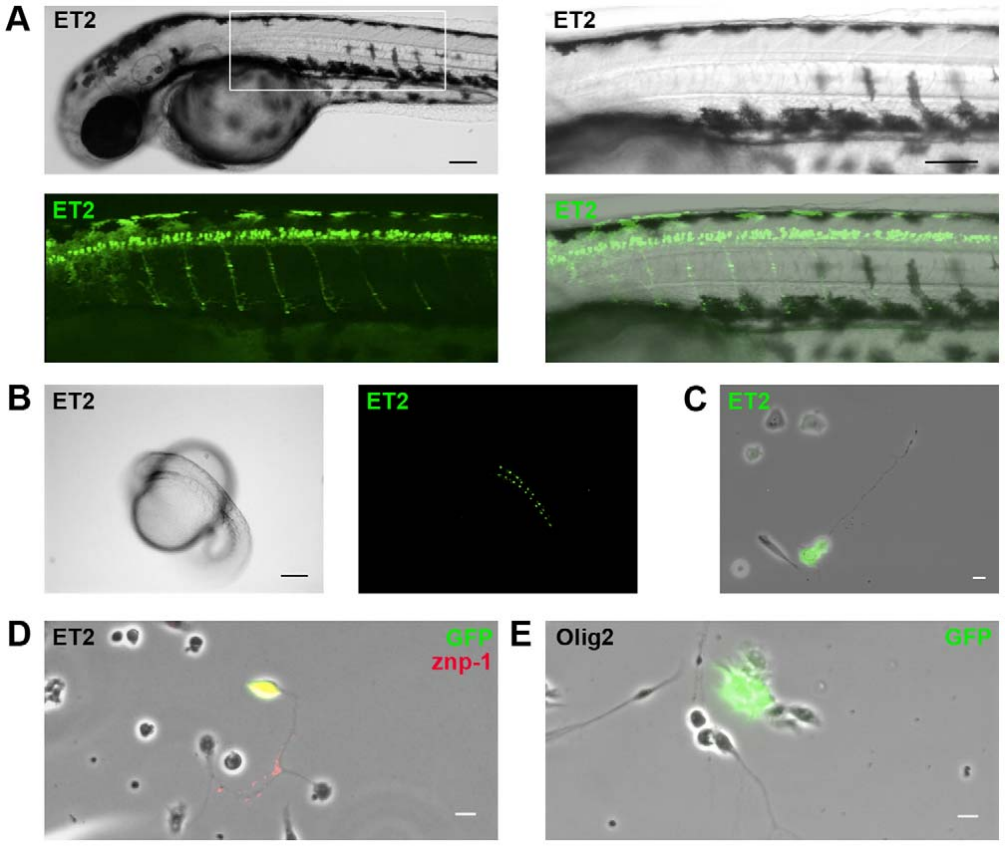
Transgenic neuron culture reveals presence of motor neurons and glial cells. (A) Mn2Et motor neuron reporter line zebrafish at 48 hpf express GFP in spinal cord motor neurons that innervate axial trunk muscles, and at (B) 20 hpf express GFP in paired spinal cord motor neurons. (C) Cultured spinal cord neurons from mn2Et reporter line zebrafish express GFP *in vitro.* (D) Immunostaining for primary motor neuron antigen znp-1 and GFP confirms presence of motor neurons in mn2Et spinal cultures. (E) Immunostaining for GFP confirms presence of glial cells in Tg(olig2:EGFP) oligodendrocyte precursor reporter line spinal cultures. Scale bars, 50 µm (A) and 10 µm (B, C, D).

We used the zebrafish reporter line mn2Et with GFP-labeled motor neurons [46] to further characterize the primary spinal neuron cultures. This transgenic line contains a transposon insertion into the gene for poly(ADP-ribose) glycohydrolase and expresses GFP in caudal primary motoneuron cell bodies and axonal trajectories [46]. At 2 days post fertilization (dpf), bright motor neurons can be visualized in mn2Et zebrafish *in vivo,* with axons extending ventrally from cell bodies in the dorsal spinal cord to innervate axial muscles (Fig. 2A). Paired primary motor neurons in the developing spinal cord began expressing GFP at the 18 somite stage, and exhibited bright GFP intensity at the 20 somite stage (Fig. 2B). Live-cell fluorescence imaging of spinal cultures from mn2Et zebrafish at the 20 somite stage revealed the presence of GFP-positive motor neurons (Fig. 2C). These motor neurons extended long axons and GFP fluorescence intensity increased steadily from 6 hr to 12 hr in culture (data not shown). Immunostaining for GFP and znp-1, a primary motor neuron specific antigen, on spinal cultures from mn2Et reporter zebrafish showed doubly labeled neurons (Fig. 2D), thus validating that fluorescent cells seen *in vitro* are the same population of fluorescent motor neurons detected *in vivo*. Immunostaining for GFP on spinal cultures from a zebrafish oligodendrocyte precursor cell reporter line, Tg(olig2:EGFP); [47], confirmed the presence of glial cells in the primary cell cultures (Fig. 2E).

**Figure 3:**
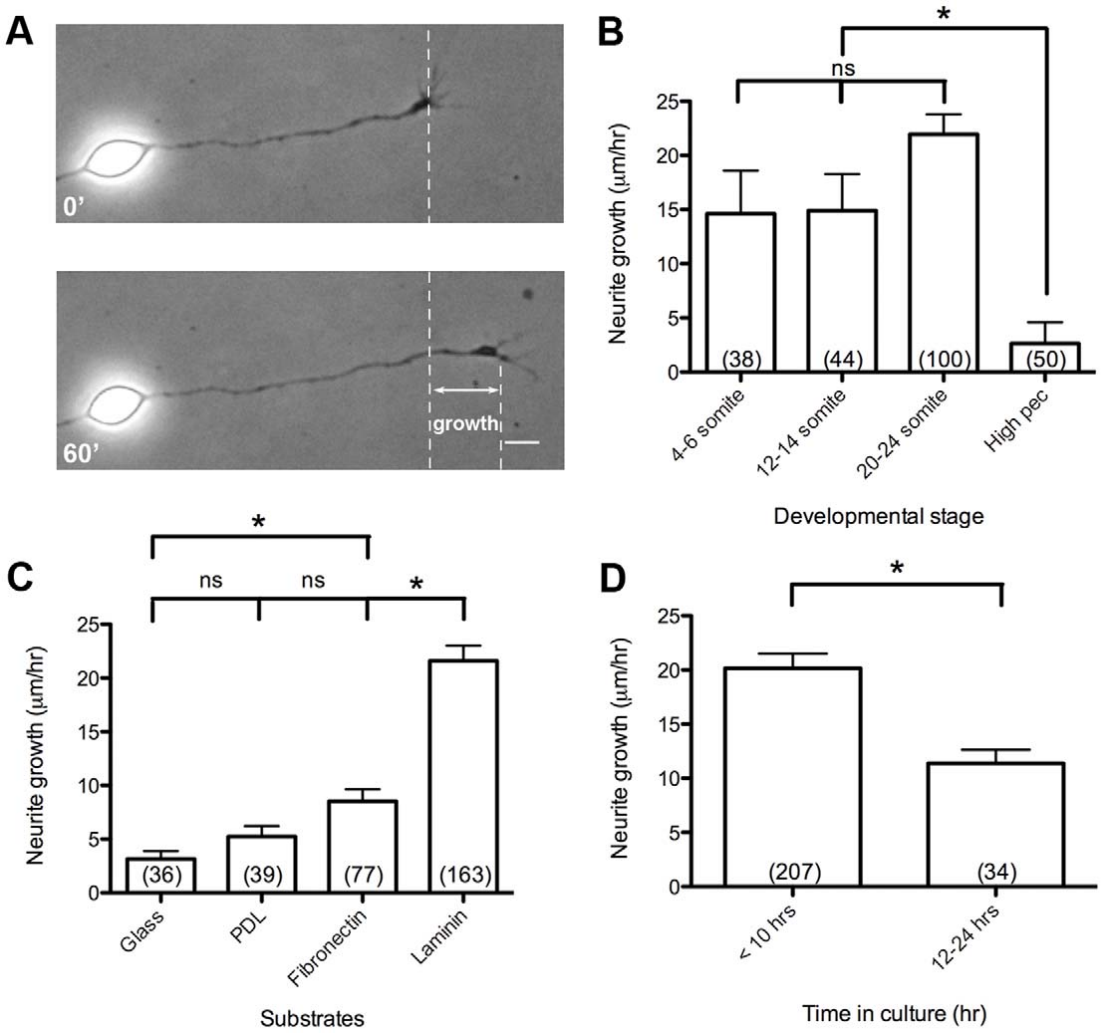
Functional growth characteristics of zebrafish spinal neurons. (A) Diagram of outgrowth assay to measure neurite growth over 1 hr. (B) Spinal neuron growth rates from embryos at 4–6 somite, 12–14 somite, 20–24 somite, and high pec stages. (C) Spinal neuron growth rates on different substrates, and (D) before 10 hr and between 12–24 hr in culture on laminin. Scale bar, 5 µm. *, p<0.05; ns, not significant.

To assess the growth potential of cultured spinal neurons at different developmental stages, we performed functional assays of neurite extension *in vitro* (Fig. 3A). Measuring the rate of neurite extension during a 1 hr time period revealed that neurons isolated from zebrafish embryos at the 20–24 somite stages had the fastest intrinsic growth rate in culture with a mean of 21.94±1.86 µm hr^−1^ s.e.m. (Fig. 3B). Neurons from younger 4–6 somite and 12–14 somite stage zebrafish, had a slower ∼15 µm hr^−1^ mean growth rate, although the difference did not reach statistical significance. Neurons from older embryos at the high pec stage, 42 hr post fertilization (hpf), had the slowest mean growth rate of 2.64±1.95 µm hr^−1^ s.e.m.

We next explored the neurite growth potential of zebrafish primary spinal neurons on different biologically relevant substrates (Fig. 3C). The mean neurite growth rate was highest, 21.6±1.42 µm hr^−1^ s.e.m, on the physiological extracellular matrix component laminin, compared to growth rates on fibronectin, poly-D-lysine, and glass. This is consistent with reports in the literature for *Xenopus* [48], chick [49], mouse [50], and rat neurons [51]. Moreover, after 3 hr in culture, neurons plated on laminin had extended neurites with a length at least 10 times the diameter of the cell body. In contrast, for neurons plated on fibronectin, poly-D-lysine, and glass, the extension of long neurites was delayed until 6 hr in culture. Spinal neurons cultured on laminin survived for at least 24 hr, but the growth rate diminished to a mean of 11.39±1.24 µm hr^−1^ s.e.m. (Fig. 3D) after 12 hr.

### Primary cell culture of forebrain, hindbrain, and retinal neurons

**Figure 4:**
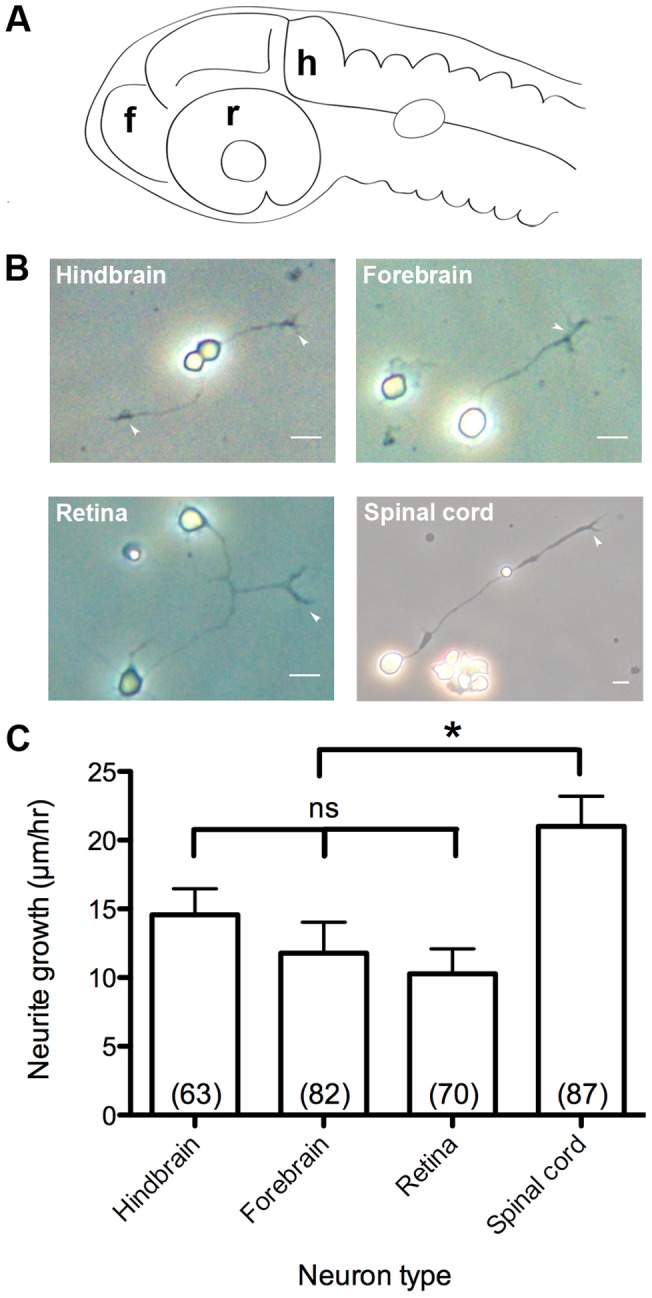
Primary culture of CNS neuron classes and growth rates *in vitro*. (A) Diagram of CNS neuron harvest sites. f, forebrain. h, hindbrain. r, retina. (B) Dissociated primary cultures of CNS neurons. Arrowheads: growth cones at axon tips. (C) Growth rates of CNS neurons. Scale bar, 10 µm. *, p<0.05; ns, not significant.

At the high pec stage, neural structures predominate in the head, and brain walls thicken substantially. We were able to dissect high pec embryos to obtain forebrain, hindbrain, and retinal (Fig. 4A) neuron primary cell cultures. These dissociated neurons appeared healthy and actively extended long processes on laminin coated coverglass (Fig. 4B). The mean growth rates of forebrain, hindbrain, and retinal axons were 11.8±2.23 µm hr^−1^, 14.6±1.89 µm hr^−1^ and 10.3±1.82 µm hr^−1^ s.e.m., respectively, with no significant differences between the groups (Fig. 4C). These growth rates were all significantly less than the mean growth rate of neurites from spinal cord neurons (21.0±2.20 µm hr^−1^) cultured under identical conditions.

### Functional outgrowth responses of cultured spinal neurons

**Figure 5:**
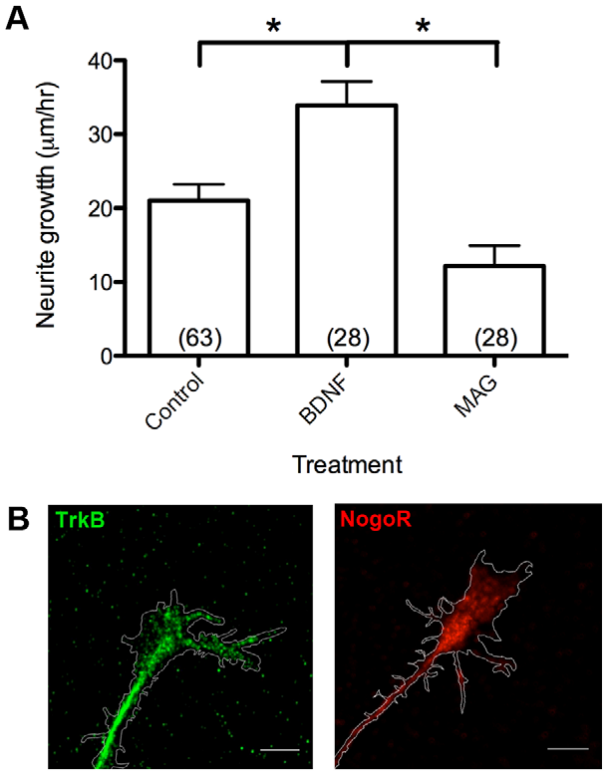
Positive and negative mammalian cues regulate axonal outgrowth. (A) Treatment with BDNF promotes while MAG inhibits axon outgrowth. (B) Staining with antibodies to mammalian BDNF receptor TrkB and MAG receptor NgR reveals high immunoreactivity at spinal neuron growth cones. Scale bar, 5 µm. *, p<0.05.

To establish and validate our primary zebrafish culture as a tool for axon guidance studies, we first measured live-cell outgrowth responses to important guidance cues in zebrafish spinal neurons *in vitro*. We performed growth assays during uniform treatment with mammalian BDNF (150 ng ml^−1^) or mammalian MAG (1 µg ml^−1^) in the bath. Cultured zebrafish spinal neurons responded positively to BDNF with increased neurite outgrowth (mean 33.9±3.20 µm hr^−1^ s.e.m.; Fig. 5A). In contrast, neurite outgrowth decreased in the presence of MAG (mean 12.2±2.75 µm hr^−1^ s.e.m.; Fig. 5A). These results are consistent with the notion that these neurons express functional receptors of BDNF and MAG, such as TrkB and Nogo receptor (NgR), respectively [43], [52]. Immunostaining of fixed zebrafish spinal neurons in dissociated culture with anti-TrkB and anti-NgR antibodies that have been demonstrated previously to recognize mammalian TrkB and NgR, respectively, indicated enrichment of presumed receptors on neuronal cell bodies (Supporting Figure S2) and growth cones (Fig. 5B), consistent with what has been previously reported for TrkB [53], [54] and NgR [55].

**Figure 6:**
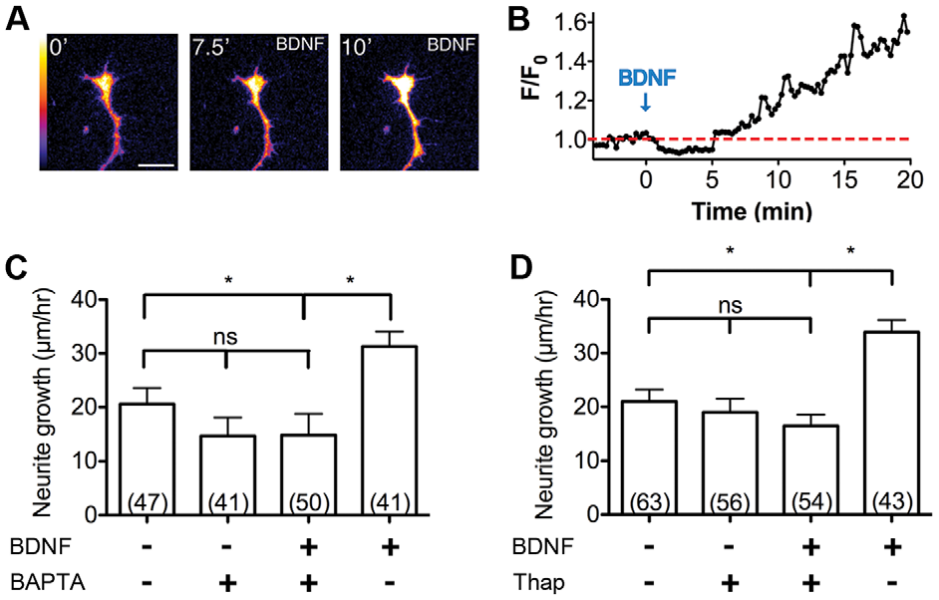
BDNF stimulated outgrowth is Ca^2+^ dependent and requires Ca^2+^ release from internal stores. (A) Bath application of BDNF elicits a strong Ca^2+^ signal in zebrafish growth cones (pseudocolored according to scale on left, with blue  =  lower [Ca^2+^] and white  =  higher [Ca^2+^]), quantified in (B). Treatment with both (C) BAPTA-AM, a cytoplasmic Ca^2+^ buffer, and (D) thapsigargin, which depletes intracellular Ca^2+^ pools, abolish BDNF stimulated outgrowth. Scale bar, 5 µm. *, p<0.05; ns, not significant.

Elevation of cytoplasmic Ca^2+^ concentration is a key mediator of BDNF/TrkB signaling during axon elongation and growth cone guidance [56], [57], [58]. We therefore tested whether BDNF induces local Ca^2+^ signals in zebrafish spinal neuron growth cones. Live-cell Ca^2+^ imaging with the membrane permeable Ca^2+^-sensitive fluorescence indicator Fluo-8H revealed the intrinsic Ca^2+^ level in the growth cone during basal outgrowth (Fig. 6A, B). Uniform bath application of BDNF induced a rise in Ca^2+^ concentration (>10% fluorescence increase) in the growth cone by 7.5 min that persisted for at least 12.5 min (Fig. 6A, B; Movie S2).

We next tested the functional role of Ca^2+^ signaling in BDNF induced axonal outgrowth. Buffering intracellular Ca^2+^ by loading zebrafish spinal neurons with the membrane permeable chelator BAPTA-AM in the presence of nominal Ca^2+^-containing media [29] caused a small but statistically insignificant reduction in the mean neurite growth rate (to 19.0±2.54 µm hr^−1^ s.e.m.) compared to untreated controls (20.1±2.26 µm hr^−1^ s.e.m.; Fig. 6C). Significantly, this Ca^2+^ buffering condition completely blocked the increase in mean growth rate normally induced by BDNF treatment, 16.45±2.01 µm hr^−1^ s.e.m. vs. 34.0±2.25 µm hr^−1^ s.e.m., respectively (Fig. 6C). Moreover, treatment with thapsigargin to deplete the internal pool of Ca^2+^ from the endoplasmic reticulum abolished BDNF-dependent outgrowth without affecting the basal growth rate (Fig. 6D). Altogether, these results implicate a requirement for Ca^2+^ release from internal stores for BDNF-induced outgrowth.

### Spinal neurons are attracted to a gradient of BDNF *in vitro*

**Figure 7:**
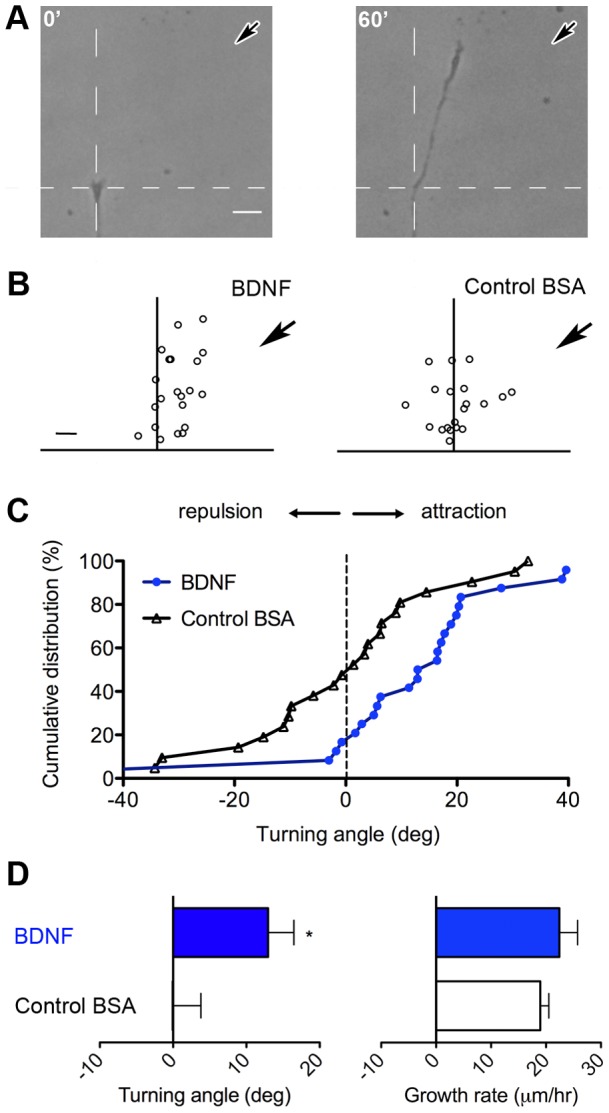
Zebrafish spinal neurons are attracted to an exponential gradient of BDNF. (A) Representative depiction of zebrafish axon turning to a BDNF gradient *in vitro*. (B) Final growth cone endpoints of turning assays. (C) Cumulative distribution and (D) mean of all turning angles to a BDNF gradient vs. control solution. Application of BDNF gradient causes no change in neurite growth rate. Scale bar, 10 µm. *, p<0.05.

Because BDNF transcripts are present throughout the zebrafish nervous system during embryonic development [59], and BDNF is a secreted chemoattractant that elicits positive growth cone chemotaxis in other model systems *in vitro* [30], [60], we tested its utility for a quantitative growth cone turning assay in zebrafish spinal neurons grown on laminin coated coverglass. Applying a microscopic gradient of BDNF by pulsatile ejection from a calibrated micropipette positioned at a 45° angle with respect to the initial direction of axon extension (see Methods) induced significant growth cone chemoattraction on laminin compared to a control solution (Fig. 7A, B, C, D). The mean neurite growth rate remained unaffected by the gradient application (Fig. 7D). Thus, even the smaller diameter zebrafish growth cones, relative to larger growth cones from the rodent and *Xenopus* CNS [61], [62], can sense and navigate up a concentration gradient of BDNF and are amenable to the quantitative turning assay *in vitro*.

## Discussion

Our findings here establish a robust embryonic zebrafish primary culture system for dissociated central nervous system neurons, including forebrain, hindbrain, retinal, and spinal cord cells, that may contribute to the field of developmental neuroscience and axon guidance. Since young navigating axons experience a complex combinatorial milieu of cues during accurate pathfinding, being able to apply and study the functional effects of one or a select pattern of axon guidance factors of interest in culture is a powerful starting point. Moreover, with the ever increasing diversity of transgenic zebrafish lines being generated, the ability to study the basis of *in vivo* neurite misrouting phenotypes in a well-defined *in vitro* system will provide molecular insights into defining the underlying guidance systems and signaling mechanisms involved.

### Utility of the zebrafish primary neuron culture system

Our approach uses basic microdissection for neural tissue extraction, and requires only standard laboratory equipment and minimal reagents. The procedure takes less than 1 hr from start of dissection to completion of tissue culture. The enzymatic and mechanical dissociation step is key to ensure neuron adherence to the coverglass in culture. The neuron cultures can be grown on the benchtop at room temperature, which negates the need for incubators with precise environmental controls. Our studies revealed that cells grow faster on physiologically relevant extracellular matrix than plain glass. Neurons plated on extracellular matrix coated coverglass are fully adherent after 2 hr, and long neurites can be seen on laminin by 6 hr. Coating coverglass with extracellular matrix is a simple and relatively quick procedure, and pre-coated coverglass are commercially available. Of importance to axon guidance studies, by using a pulled glass pipette to plate down cells, we are able to create organized lines of neuron cell bodies on the coverglass, resulting in 60–80 growth cones from 2 spinal cords per culture dish that are relatively isolated (defined as at least 200 µm away) from other cell bodies. This enables growth assays (Fig. 3, 4, 5), functional turning assays (Fig. 7), and high-resolution second messenger signaling imaging (Fig. 6) of individual growth cones.

Our spinal neuron culture technique generated a mixed population of neurons in the culture dish, including commissural interneurons, motor neurons, and glial cells. Using transgenic reporter zebrafish, for example the mn2Et motor neuron reporter line, individual subsets of neurons can be identified *in vitro* for functional studies. Developmental stage of the embryo and adhesion to extracellular matrix components had a significant effect on the growth potential of neurons, with spinal neurons from 20–24 somite stage embryos having the fastest growth rate in culture on laminin-coated coverglass.

Established zebrafish explant culture methods from retina [32] and hindbrain [33] allow study of these neuron populations *in vitro*, and here we introduce a novel technique to generate dissociated neuron cultures from head CNS structures such as the hindbrain, forebrain, and retina. Previously reported outgrowth rates from retinal ganglion cell explants are about 15±3 µm s.e.m during a 1.5 hr period [32], which is comparable to our dissociated retinal neuron growth rate of 10.3±1.82 µm hr^−1^ s.e.m. It is remarkable that the growth rates provided by these distinct methods are so similar due to the differences in the age of zebrafish larvae –48 hpf vs. 42 hpf in the present study – and neuron composition – a more homogeneous retinal ganglion cell population vs. a likely mixed population of retinal neurons in our dissociated cultures.

The zebrafish model system is a relatively new educational tool [63], [64] but offers exciting opportunities for improving experiential, laboratory based scientific learning and for connecting neurobiologists with the general community. Zebrafish are cost effective and the housing facilities are easy to maintain, making this model system attractive even for educational programs with limited laboratory budgets. A few adult pairs can reliably yield high numbers of translucent, relatively large embryos with fast and predictable temperature-dependent development. In addition to wild-type zebrafish, a growing number of mutant and transgenic lines are widely available for research and educational purposes. Thus, our relatively simple neuron culture system is amenable to introducing concepts of *in vitro* research in the classroom. With these cultures, students can pursue hypothesis driven science exploration and learn essential laboratory skills like microdissection, phase contrast microscopy, and image analysis using NIH ImageJ or other advanced image processing software.

### Regulation of axon growth and guidance by BDNF

Cultured zebrafish spinal neurons express the BDNF receptor TrkB, which is known to play a critical role in nervous system development in other species [52], [60]. To our knowledge, this is the first report that an environmental gradient of BDNF induces zebrafish growth cone attraction. Although BDNF gene expression begins at the time of neurogenesis in *Xenopus*, higher vertebrates, and mammals, levels are low in the developing regions of the CNS and increase as these regions mature [59], [65]. In contrast, in zebrafish BDNF transcripts are present at high levels in the developing neural tube, with progressive restriction of BDNF expression correlating with specialization of cell types, resulting in only small subsets of BDNF expressing cells by 4 dpf associated with epithelial, cartilaginous, and vascular structures [66]. Thus in zebrafish, BDNF may be a physiologically relevant spinal neuron guidance cue whose expression is restricted to the time period of initial nervous system wiring. Furthermore, recent reports have implicated decreased BDNF expression at 24 hpf as a major contributor to the neurodevelopmental defects seen in huntingtin knockdown zebrafish [67].

Application of BDNF promotes outgrowth of spinal neurons and induced a rapid rise of Ca^2+^ within the growth cone by 5 min. This fast onset Ca^2+^ signal appears to be necessary for BDNF induced outgrowth. The stimulatory effect of BDNF is abolished by buffering intracellular Ca^2+^ with BAPTA-AM or by depleting internal Ca^2+^ stores with thapsigargin. It is likely that signaling pathways involving Ca^2+^ influx and the PI(_3,4,5_)P3/Akt/TRPC cascade [30] – mechanisms largely conserved from amoeboids to higher vertebrates – may also play a role in BDNF induced attraction in zebrafish embryos. Interestingly, our study shows that restricting intracellular Ca^2+^ concentration and inhibiting Ca^2+^ release from internal stores have no affect on basal growth rate of zebrafish spinal neurons during the experimental window.

The zebrafish primary neuron cultures were grown for several hr in culture media containing 0.4% penicillin/streptomycin, and were switched into culture media without penicillin/streptomycin for a 30-min pre-period prior to beginning functional growth and turning assays. We noted that during this pre-period, the neurons exhibit a burst of rapid growth (data not shown), which returns back to a slower rate of growth by 30 min. Certain antibiotic classes are potent transient receptor potential channel blockers that inhibit Ca^2+^ influx [68]. Thus, the rapid removal of antibiotics from the culture media may relieve inhibition of transient receptor potential channels. How such disinhibition might regulate intracellular Ca^2+^ concentration to promote outgrowth remains an interesting area for future investigation.

### Axon growth inhibition by MAG

In contrast to mammals, the zebrafish central nervous system regenerates robustly after injury. Fish retinal ganglion cell growth cones collapse upon contact with mammalian oligodendrocytes, but can grow normally with fish oligodendrocytes [69], [70]. Our finding that mammalian MAG inhibits zebrafish spinal neuron outgrowth raises the question of whether mammalian MAG binds the fish NgR or another receptor complex, and whether this binding elicits activation of a distinct signal transduction pathway. It is likely that the absence of inhibitory effects of endogenous fish MAG on fish neurons is partly responsible for not only the robust regeneration seen in the zebrafish model system, but also the plasticity and accurate pathfinding seen in regenerating neurites. Thus, the novel *in vitro* culture system utilized in this present study may prove useful for defining the mechanistic differences underlying robust versus limited regeneration in zebrafish and mammals.

## Materials and Methods

### Homology of axon guidance pathways

Homologs were identified using HomoloGene, and all sequence data was obtained from the National Center for Biotechnology Information (NCBI). All human-zebrafish pairwise sequence alignments were performed using NCBI BLAST (blastp) method with default parameters.

### Primary cell culture of zebrafish spinal neurons

All of the animal work in this study conformed to animal protocol #A8410, reviewed and approved by the Mayo Clinic Institutional Animal Care and Use Committee (IACUC) on April 5^th^, 2010. Cultures of spinal neurons were prepared from 4 somite (11.5 hpf) to high pec (42 hpf) stage Segrest wild type *Danio rerio* embryos. Using a flame polished broken glass pipette, embryos were immersed in 70% EtOH for 5 sec for sterilization and transferred through two washes of sterile 0.5X E2 embryo media (as described in [71]). The chorion was removed and the embryos were transferred to a fresh dish of sterile 0.5X E2 embryo media. Fine forceps were used to first clip off the tail bud and structures anterior to the first somite, followed by separation of the body from both the yolk sac and the Kupffer's vesicle. The developing skin was removed in two large pieces. The neural tube tissue was transferred to the dissociation medium (in mM: 115 NaCl, 2.5 KCl, 0.4 EDTA, 8 Hepes, 0.05% (m/v) trypsin (Worthington), pH 7.5) for 30 min. Using a 10 µL pipette, the neural tub tissue was then transferred into a microcentrifuge tube containing 5 µL of culture medium (85.9% (v/v) Leibovitz medium (GIBCO) containing 2% (v/v) fetal bovine serum, 0.4% (v/v) penicillin/streptomycin, and 12.5% (v/v) saline solution (in mM: 10 D-glucose, 5 Na-pyruvate, 1.26 CaCl2, and 32 Hepes), pH 7.5). Mechanical dissociation was performed using a 10 µL pipette and triturating up and down 50 times. The cell suspension was drawn up in a pulled glass pipette with an opening diameter of 100 µm and plated down in parallel thin lines onto coverglass immersed in 1 mL culture medium. Coverglass was either plain flame polished glass, glass coated with poly-D-lysine (Sigma, 0.5 mg ml^−1^), glass coated with poly-D-lysine followed by fibronectin (Sigma, 20 µg ml^−1^), or glass coated with poly-D-lysine followed by laminin (Invitrogen, 20 µg ml^−1^). Cultures were maintained on the benchtop at 22°C. Experiments were performed 6–24 hr after plating, in culture medium without antibiotics at 28.5°C.

### Primary cell culture of zebrafish forebrain, hindbrain, and retinal neurons

Cultures of forebrain, hindbrain, and retinal neurons were prepared from high pec (42 hpf) stage zebrafish embryos. After fully dechorionating, embryos were transferred to 0.5X E2 embryo media +0.04% (m/v) tricaine methanesulfonate (MS-222) and decapitated. Forebrain tissue was isolated by peeling back the developing skin covering the head, and extracting the telencephalon rostral to the eyes. Hindbrain tissue was isolated by identifying the border of the cerebellar primordium, and extracting tissue for 150 µm caudally, corresponding to rhombomeres r2– r7. Retinal cells were isolated by peeling back the overlying skin and lens rudiment, and separating the retina from the underlying tissue. The tissue was then dissociated and cells were plated down on coverslips, similar to the procedure for spinal neurons as described above.

### Live-cell quantitative assay of neurite outgrowth

For measurements of neurite outgrowth, we took time-lapse phase contrast (20x) images of individual neurites over a 60 min period. For growth assays in response to BDNF or MAG, we incubated cells in culture media containing BDNF (150 ng ml^−1^) or MAG (1 µg ml^−1^). For growth assays showing the dependence of BDNF induced outgrowth on Ca^2+^, we incubated cells in nominal Ca^2+^ saline (30 nM) for 30 min consisting of 50% culture medium and 50% EGTA-buffered saline (in mM: 120 NaCl, 4.9 KCl, 2.55 MgCl2, 1.25 glucose, 5-Na-pyruvate, 4 Hepes, 0.65 EGTA), then treated with 1 µM BAPTA-AM for an additional 30 min while maintaining extracellular Ca^2+^at 30 nM. We removed remaining extracellular BAPTA-AM by consecutive washes in reduced Ca^2+^ saline and then incubated cells for an additional 20 min equilibration period prior to BDNF treatment. Thapsigargin (100 nM) was added to the culture media for 20 min prior to BDNF treatment and start of growth assays.

Outgrowth assays were performed with neuron cultures on Zeiss Axiovert 40 microscopes equipped with Ludl Electronic Products (Hawthorne, NY, USA) BioPoint 2 motorized stages. Images were taken with 20x objectives and analyzed for growth using the ImageJ software. Only the longest neurite or branch of an individual neuron was measured, and only axons >10 times the diameter of the neuron body were included in the analysis. Neurites from at least 3 independent culture dishes were used per experimental condition.

### Immunocytochemistry, fluorescence microscopy and image analysis on fixed cells

All neuron cultures were chemically fixed in a cytoskeleton stabilizing buffer containing 4% paraformaldehyde and 0.01% glutaraldehyde for 20 min. All blocking and immunolabeling steps were performed in antibody buffer (in mM: 120 NaCl, 2.2 KCl, 2 CaCl2, 1 MgCl2, 5 Hepes, 2 Na-pyruvate, pH 7.5) containing 5% goat serum. We labeled cells using a polyclonal antibody to TrkB (rabbit anti-TrkB, Novus, 10 µg mL^−1^), polyclonal antibody to NgR (rabbit anti-NgR, Santa Cruz Biotechnology, 2 µg mL^−1^), polyclonal antibody to GFP (rabbit anti-GFP, Abcam, 10 µg mL^−1^), and monoclonal antibody to znp-1 (mouse anti-znp-1, Developmental Studies Hybridoma Bank, 5 µg mL^−1^). Alexa-dye labeled secondary antibody conjugates (Invitrogen) were used at 2 µg mL^−1^ and imaging was performed on confocals LSM 5-Live and LSM 780.

### Live zebrafish imaging

Embryos younger than 24 hpf were imaged in 2.5% methylcellulose with an inverted Zeiss Apotome. Embryos older than 24 hpf were imaged according to the Specimen in a Corrected Optical Rotational Enclosure protocol (as described in [72]) combined with 5x and 10x water immersion lenses using an inverted Zeiss Apotome.

### Functional assay of growth cone turning

To generate exponential gradients, we positioned calibrated micropipettes filled with BDNF (50 ug mL^−1^) or control saline + BSA vehicle (0.1%) 100 µm away from the growth cone at a 45° angle to the initial direction of neurite extension as described previously in [24], [73]. A standard pressure pulse was generated by a picospritzer so that the concentration at the growth cone was 1000 fold less than the concentration in the pipette. A CCD camera recorded phase images at the start of the turning assay and after 1 hr at the end of the turning assay. The turning angle was determined as described previously in [24], [73].

### Live-cell Ca^2+^ imaging

Cultured zebrafish spinal neurons were loaded with Fluo-8H (2 µM, AAT Bioquest) for 30 min at 22°C. Ca^2+^ imaging was performed within 45 min of dye loading using a Zeiss ratio imaging system equipped with a 100× objective and Hamamatsu EM-CCD camera. Excitation was at 488 nm and the Fluo-8H emission signals were collected at 500–560 nm. Fluorescence images were captured at 15 sec intervals. The mean fluorescence intensity above background threshold was measured over the entire growth cone. The fluorescence at each time point was normalized to the average fluorescence ratio that was measured during a 5 min baseline period prior to treatment with BDNF (150 ng mL^−1^).

### Statistical analysis

All data was analyzed with GraphPad Prism software (v5a). All outgrowth data was normally distributed (analyzed with D'Agostino – Pearson omnibus normality test), and was assessed with repeated-measures one-way analysis of variance and two-tailed Student's t-tests. Two-tailed Student's t-tests were used to analyze Ca^2+^ imaging data. Statistical comparisons involving turning assay experiments used the Mann-Whitney U test due to the nonparametric distribution of the data.

## Supporting Information

Figure S1 **Dissociated spinal neuron cultures show neurons and a mixed population of other cell types, morphologically resembling myoblasts and glial cells. Arrows: myoblasts. Scale bar, 20 µm.**
(TIF)

Figure S2 **Staining with antibodies to mammalian BDNF receptor TrkB and MAG receptor NgR reveals high immunoreactivity at spinal neuron cell bodies.**
(TIF)

Movie S1 **Time-lapse differential interference contrast microscopy images of a zebrafish spinal neuron growth cone in culture.** Time-lapse differential interference contrast microscopy images of a zebrafish spinal neuron growth cone in culture showing dynamic protrusion and retraction of filopodia and lamellipodia. Time in sec.
(MOV)

Movie S2 **Biosensor imaging of a calcium signal induced by uniform application of BDNF in a zebrafish spinal neuron growth cone in culture.** Time-lapse images of a zebrafish spinal neuron growth cone in culture loaded with fluorescent Ca^2+^ sensor Fluo-8H (pseudocolored according to scale in Figure 6A). At time  = 0∶00 min, BDNF is applied in the bath and initiates a Ca^2+^ signal in the growth cone by 5 min. This movie corresponds to Figure 6A.
(MOV)
