# Supplementary figures and images for: Primary Neuron Culture for Nerve Growth and Axon Guidance Studies in Zebrafish (Danio rerio)
Source: PLoS One. 2013 Mar 4;8(3):e57539. doi: 10.1371/journal.pone.0057539 (PMC3587632; doi:10.1371/journal.pone.0057539)

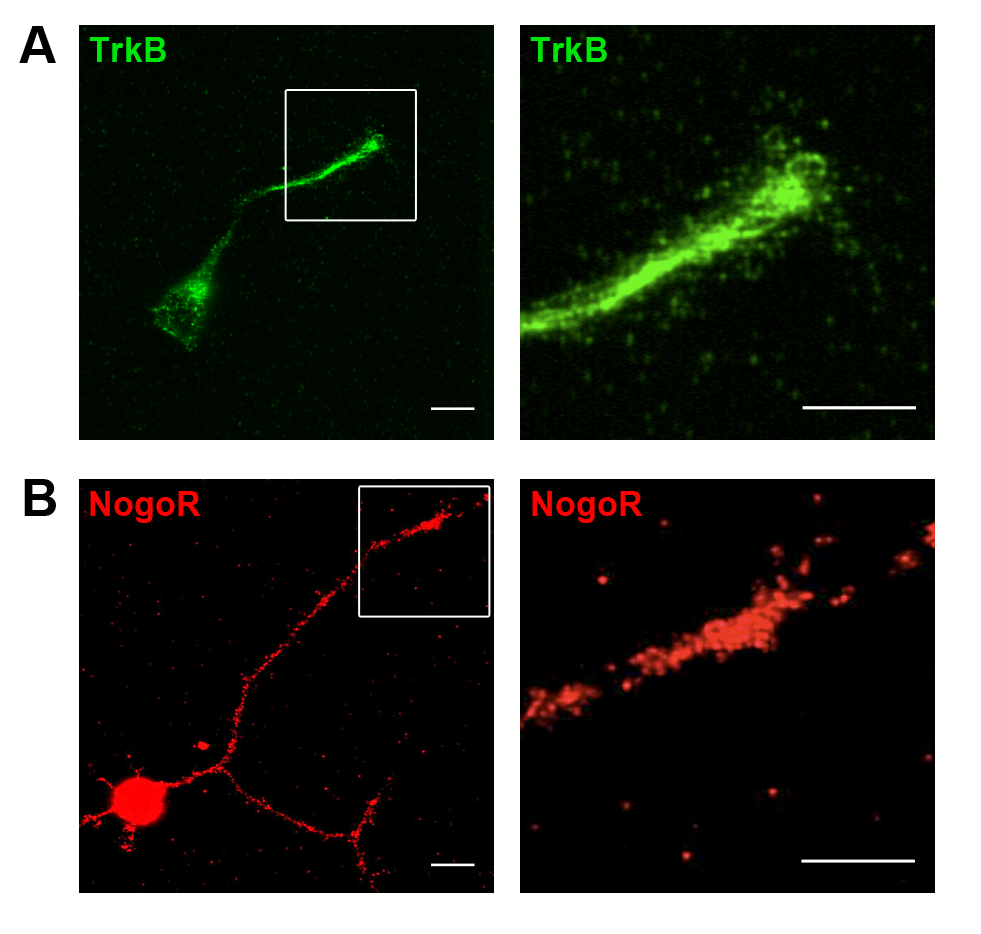

Supplement: Figure S1 — Dissociated spinal neuron cultures show neurons and a mixed population of other cell types, morphologically resembling myoblasts and glial cells. Arrows: myoblasts. Scale bar, 20 µm. (TIF) [file pone.0057539.s001.tif]

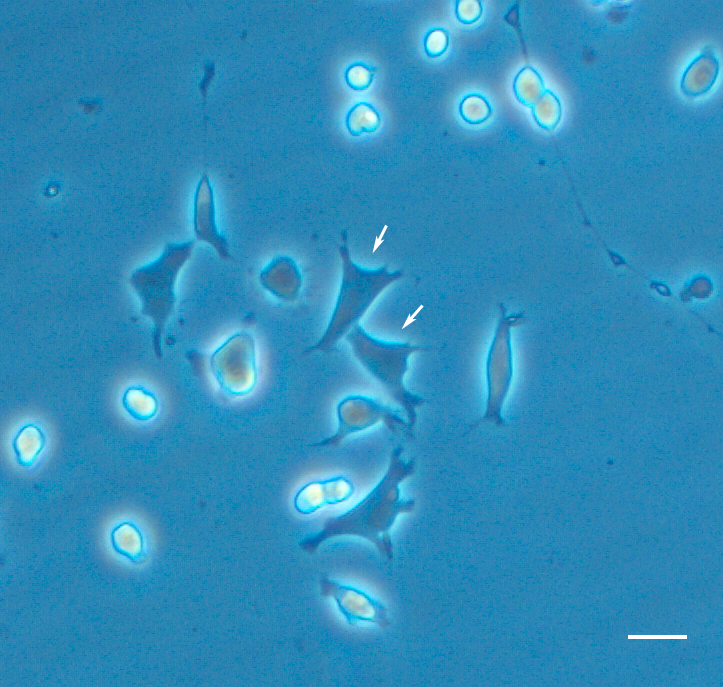

Supplement: Figure S2 — Staining with antibodies to mammalian BDNF receptor TrkB and MAG receptor NgR reveals high immunoreactivity at spinal neuron cell bodies. (TIF) [file pone.0057539.s002.tif]
